# Development of an adverse outcome pathway network for nephrotoxicity

**DOI:** 10.1007/s00204-023-03637-7

**Published:** 2024-01-10

**Authors:** D. A. Barnes, J. W. Firman, S. J. Belfield, M. T. D. Cronin, M. Vinken, M. J. Janssen, R. Masereeuw

**Affiliations:** 1 Division of Pharmacology, Utrecht University, Utrecht Institute for Pharmaceutical Sciences, Utrecht, The Netherlands; 2 School of Pharmacy and Biomolecular Sciences, Liverpool John Moores University, Liverpool, UK; 3 Department of Pharmaceutical and Pharmacological Sciences, Entity of In Vitro Toxicology and Dermato-Cosmetology, Vrije Universiteit Brussel, Brussels, Belgium

**Keywords:** Nephrotoxicity, Adverse outcome pathway, Key events, Molecular initiating event, New approach methodology

## Abstract

Adverse outcome pathways (AOPs) were introduced in modern toxicology to provide evidence-based representations of the events and processes involved in the progression of toxicological effects across varying levels of the biological organisation to better facilitate the safety assessment of chemicals. AOPs offer an opportunity to address knowledge gaps and help to identify novel therapeutic targets. They also aid in the selection and development of existing and new in vitro and in silico test methods for hazard identification and risk assessment of chemical compounds. However, many toxicological processes are too intricate to be captured in a single, linear AOP. As a result, AOP networks have been developed to aid in the comprehension and placement of associated events underlying the emergence of related forms of toxicity—where complex exposure scenarios and interactions may influence the ultimate adverse outcome. This study utilised established criteria to develop an AOP network that connects thirteen individual AOPs associated with nephrotoxicity (as sourced from the AOP-Wiki) to identify several key events (KEs) linked to various adverse outcomes, including kidney failure and chronic kidney disease. Analysis of the modelled AOP network and its topological features determined mitochondrial dysfunction, oxidative stress, and tubular necrosis to be the most connected and central KEs. These KEs can provide a logical foundation for guiding the selection and creation of in vitro assays and in silico tools to substitute for animal-based in vivo experiments in the prediction and assessment of chemical-induced nephrotoxicity in human health.

**Supplementary Information:**

The online version contains supplementary material available at 10.1007/s00204-023-03637-7.

## Introduction

An adverse outcome pathway (AOP) is a scientific framework which utilises the available mechanistic information related to a toxicological response to provide a comprehensive description of sequential biological events, resulting in an adverse effect following exposure to a stressor (Ankley et al. 2010). Since its establishment in 2010, the concept of the AOP has gained significant attention within chemical risk assessment communities (Marx-Stoelting et al. 2023). As such, it presents a valuable means towards the advancement of in silico tools such as quantitative structure–activity relationships (QSARs) and innovative in vitro toxicity screening tests (Cronin and Richarz 2017; OECD 2017; Vinken 2013). AOPs comprise a series of causal connections across varying levels of the biological organisation—originating from a molecular perturbation triggered by a stressor, known as the molecular initiating event (MIE), and proceeding through a sequence of intermediate key events (KE), before culminating in the adverse outcome (AO) (OECD 2018a; Vinken et al. 2017). These KEs are interconnected by key event relationships (KERs), which represent the causal link between perturbation in one and emergence of another. AOPs have gained importance as a valuable tool for the development and implementation of in vitro and in silico testing strategies for assessing toxicity in recent years, helping to provide a novel understanding of regulatory-relevant in vivo outcomes (Kleinstreuer et al. 2016).

The Organisation for Economic Cooperation and Development (OECD) has recognised the benefits of AOPs for improving the efficiency of chemical safety assessment and has been actively promoting their development since 2012 (OECD 2017). As part of a programme to facilitate their advancement, the OECD established the AOP Knowledgebase (https://aopkb.oecd.org/) as a centralised repository for AOP-related information (OECD 2018a). Included within this knowledgebase, the AOP-Wiki (https://aopwiki.org/) offers a transparent platform for the collaborative development of qualitative AOP descriptions, aiming to promote their incorporation into risk assessment strategies and to facilitate the reuse of mechanistic toxicological knowledge. As of May 2023, there have been over 400 AOPs and 2000 KEs listed on the AOP-Wiki. AOPs and the mode-of-action framework are similar in that they describe molecular mechanisms that result in AOs. However, unlike the mode-of-action framework, AOPs offer a purely dynamic and biological perspective to describe a toxicological process and are, therefore, chemical-agnostic (Vinken et al. 2017). This means that an AOP can be linked to any stressor that is bioavailable at the target site and possesses the characteristics needed to trigger the connected MIE.

Currently, a single AOP is designed as a practical unit to describe causal links between KEs, rather than a comprehensive biological representation of all possible molecular, biochemical, and physiological components involved in toxicological processes. As a result, individual AOPs are often developed as “linear” constructs, without any converging or diverging pathways connected to them. Therefore, a single AOP may not encompass all events that could contribute to the relevant adverse effect being described (Sewell et al. 2018). Most toxicologically relevant processes and real-world scenarios would require multiple AOPs to describe and predict given AOs, particularly when concerning mixtures or the multigenerational effects of chemicals (Groh et al. 2015; Villeneuve et al. 2014). As such, the future utilisation of AOPs for risk assessment would benefit from the development of multiple interacting pathways and networks. While individual AOPs only offer a linear description of biological processes, their merging via common shared KEs and KERs can provide an improved description of the biological complexities inherent to the system studied and, as such, offer additional value. As such, some authors have already begun submitting individual AOPs as networks instead of the typical linear format, e.g. AOP 138.

With the development of greater numbers of AOPs, it is inevitable that networks will emerge. These have the potential to consolidate a range of information, previously viewed in isolation, on the potential AOs that may arise from a disturbance of a biological pathway. AOP networks consist of sets or groupings of interrelated AOPs that share one or more KEs in common (Villeneuve et al. 2014). Alternatively, AOP networks can also be formed by AOPs that either diverge from a single MIE or converge to a single AO, even if they do not share any further intermediate KEs (Knapen et al. 2018). Combining MIEs, KEs, and AOs across various AOPs into a unified network offers a more realistic depiction of possible (bio)chemical effects. This approach provides additional insights into interactions between AOPs by presenting specific combinations or sequences of KEs, thus potentially unveiling previously undiscovered or unconsidered connections between biological pathways. In this way, AOP networks enable the visualisation and recognition of features of interest, including those KEs most central and well-connected, the points at which AOPs converge or diverge, and also the presence of positive and negative feedback loops (Knapen et al. 2018; Villeneuve et al. 2018). Furthermore, while the identification of common KEs within AOP networks can facilitate the development of assays that detect adverse effects of regulatory interest, distinctions should also be made when differentiating between generic and disease-specific KEs. A generic KE will be described in a general, abstract manner, and will be applicable either across different AOPs or in multiple instances of a single AOP. While generic KEs capture common biological responses or events across different AOPs, disease-specific KEs focus on the unique characteristics and mechanisms associated with a particular disease or AO. Disease-specific KEs are typically discovered through scientific research, epidemiological studies, or clinical observations, which each may shed light on the distinct molecular, cellular, or physiological changes that take place throughout its advancement. Notably, they can both offer valuable insights into the pathways or processes that play a crucial role in development, progression, or degree of severity. Such disease-specific KEs should be interconnected, either with generic or with other disease-specific KEs, to form a more comprehensive AOP network.

AOP networks are particularly important for addressing exposures to multiple stressors that lead to the same AO or else to individual stressors that perturb multiple MIEs. As such, they may aid in the understanding of potential interactions between co-occurring AOPs (Knapen et al. 2015; Villeneuve et al. 2014, 2018). By describing the components of an AOP (KEs and KERs) in a modular fashion, AOP networks can be constructed from individual pathways that share KEs—whereby KEs are represented as nodes and KERs as directed edges acting to link them (OECD 2018c). The tools of network science can utilise this modular format to quantitatively analyse AOP networks and identify KEs of interest (Villeneuve et al. 2018). For the modelling and prediction of nephrotoxicity, AOP networks can identify and integrate existing knowledge about the key biological events and pathways underlying kidney damage and translate this knowledge into practical applications for toxicology and risk assessment. In this study, we used an approach previously applied in AOP networks for neurotoxicity (Spinu et al. 2019) and hepatotoxicity (Arnesdotter et al. 2021)—following established guidelines for AOP network derivation, characterisation and analysis (Knapen et al. 2018; Villeneuve et al. 2018)—to identify relevant KEs that could be used to inform the establishment of a battery of in vitro and/or in silico assays for the prediction of nephrotoxicity.

## Materials and methods

### AOP network derivation

A manual search of the OECD AOP-Wiki 2.0 (https://aopwiki.org/) was conducted to identify individual AOPs concerning nephrotoxicity. Relevant information for each AOP, including KE titles, KE types (MIE, KE, AO), KERs (links between upstream and downstream KEs), KE relationship adjacency, qualitative weight of evidence (WoE), developmental stage, and OECD review and endorsement progress, was extracted and recorded in an Excel spreadsheet. This information was collected on 1 May 2023 and is provided as supplementary data in Appendix 1. The development of the nephrotoxicity AOP network followed a four-step method described previously (Arnesdotter et al. 2021; Spinu et al. 2019).

#### Step 1: Definition of purpose

The aim of this study was to identify the most central and frequently occurring KEs and KERs in an AOP network describing nephrotoxicity. This information will serve as a foundation for identifying measurable in vitro assays that can predict the harmful effects of chemicals on the kidney. The scope of this study involved examining those individual AOPs, created for nephrotoxicity, which have previously been uploaded onto the AOP-Wiki resource.

#### Step 2: Definition of criteria for AOP selection

AOPs were selected based on several criteria: their development stage, KER adjacency, and WoE assessment. The AOP development stage reflects the level of maturity of the AOP as it progresses through the OECD review process. KERs describe the connections between upstream and downstream KEs and may be considered either adjacent (direct) or nonadjacent (indirect). The WoE assessment reports the qualitative level of understanding of the KE relationships described by the individual AOP developers. Analysing these criteria can help in identifying areas of uncertainty within the network and thus may guide efforts to clarify the underlying mechanisms.

#### Step 3: Identification of appropriate AOPs from the AOP-Wiki and data curation

The process of compiling selected AOPs involved manual evaluation, followed by consolidation of the relevant information into an Excel database. In instances where multiple KEs shared one meaning, or else referred to similar processes, they were grouped and assigned a common title. Abbreviations were also utilised where appropriate. Any modifications made to the KE titles are documented within the Excel database, which is included as supplementary information (Supplementary Information, SI1).

#### Step 4: Generation and analysis of the network

The AOP network was developed using the open-source software platform Cytoscape (v. 3.9.1;https://cytoscape.org/) (Shannon et al. 2003). NetworkAnalyzer, a pre-installed Cytoscape application, was used for network analysis. Nodes within the network were manually positioned to optimise space and readability. Further annotations, including WoE, KE adjacency, and type, were added to enhance the clarity of the network's visual components. Although quantitative network analysis considers the multiple relationships which may exist between KEs as being distinct, single arrows are nevertheless used in pictorial representations.

### Network analysis

Applying directional analysis, the Cytoscape NetworkAnalyzer module was used in order to compute standard network metrics including degree, eccentricity, and betweenness centrality. With the assistance of the PesCa plugin (v. 3.0; https://apps.cytoscape.org/apps/pesca30) an additional parameter, AOP simple path occurrence (normalised), was calculated (Scardoni et al. 2015). Described by Villeneuve et al., this represents an adaptation of standard betweenness centrality, rendering it more informative within the context of the AOP network (Villeneuve et al. 2018). In brief, whereas betweenness centrality is determined through consideration of shortest-route connections linking all node pairs, the AOP simple path occurrence instead accounts only for those associating MIEs with AOs. Events scoring highest within this were considered most highly connected and influential. In-degree and out-degree counts were further used to identify convergence and divergence points, as well as overall connectivity.

## Results

### Development of the AOP network for nephrotoxicity

After manually searching the AOP-Wiki, an initial 18 individual AOPs that pertained to nephrotoxicity were identified. Following our established criteria (as described within Sect. “Network analysis”), we deemed 13 of these AOPs to be suitable for integration into the network. Five AOPs were excluded due to either reporting no adjacency metrics, being too specifically focussed on the reported AOPs or being ill-defined (an overview of both the omitted AOPs and the exclusion criteria can be found in SI1).

Table 1 gives details on the selected AOPs and their developmental stage at the time of retrieval. Inconsistencies in KE annotation have been recognised as a challenge in the development of AOP networks (Spinu et al. 2019). In this study, discrepancies in KE naming conventions were reviewed to avoid conflicting events. For instance, KEs denoting mitochondrial dysfunction, such as KE 1483 (Dysfunction, Mitochondria), KE 1968 (Increase, Mitochondrial Dysfunction), and KE 177 (N/A, Mitochondrial Dysfunction), were harmonised and pooled to ensure that different KEs describing the same processes were not treated as distinct. Details of the annotation changes are provided in the supplementary material (SI1) for reference.

**Table 1:** Individual nephrotoxicity AOPs selected for inclusion within the AOP network

ID	Title	MIE	AO	Point of contact	OECD status	Source
105	Alpha_2u_-microglobulin cytotoxicity leading to renal tubular adenomas and carcinomas (in male rat)	Increased, binding of chemicals to α2u (serum)	Increase, adenomas/carcinomas (renal tubular)	Charles Wood		https://aopwiki.org/aops/105
116	Cytotoxicity leading to renal tubular adenomas and carcinomas (in male rat)	Increase, cytotoxicity (tubular epithelial cells)	Increase, adenomas/carcinomas (renal tubular)	Charles Wood		https://aopwiki.org/aops/116
138	Organic anion transporter (OAT1) inhibition leading to renal failure and mortality	Inhibition, organic anion transporter 1 (OAT1)	Increased mortality and decline, population	Kellie Fay		https://aopwiki.org/aops/138
177	Cyclooxygenase 1 (COX1) inhibition leading to renal failure and mortality	Inhibition, cyclooxygenase 1 activity	Increased mortality and decline, population	Kellie Fay		https://aopwiki.org/aops/177
186	Unknown MIE leading to renal failure and mortality	Unknown, MIE	Increased mortality	Kellie Fay		https://aopwiki.org/aops/186
256	Inhibition of mitochondrial DNA polymerase gamma leading to kidney toxicity	Inhibition of mitochondrial DNA polymerase gamma (Pol gamma)	Occurrence, kidney toxicity	Angela Mally	Underdevelopment	https://aopwiki.org/aops/256
257	Receptor-mediated endocytosis and lysosomal overload leading to kidney toxicity	Binding of substrate, endocytic receptor	Occurrence, kidney toxicity	Angela Mally	Underdevelopment	https://aopwiki.org/aops/257
258	Renal protein alkylation leading to kidney toxicity	Alkylation, protein	Occurrence, kidney toxicity	Angela Mally	Underdevelopment	https://aopwiki.org/aops/258
284	Binding of electrophilic chemicals to SH(thiol)-group of proteins and /or to selenoproteins involved in protection against oxidative stress leads to chronic kidney disease	Binding, thiol proteins/selenoproteins involved in protection against oxidative stress	Chronic kidney disease	Frederic Y. Bois		https://aopwiki.org/aops/284
384	Hyperactivation of ACE/Ang-II/AT1R axis leading to chronic kidney disease	Hyperactivation of ACE/Ang-II/AT1R axis	Chronic kidney disease	Young Jun Kim		https://aopwiki.org/aops/384
413	Oxidation and antagonism of reduced glutathione leading to mortality via acute renal failure	Oxidation, glutathione	Increased kidney failure and mortality	Zarin Hossain		https://aopwiki.org/aops/413
437	Inhibition of mitochondrial electron transport chain (ETC) complexes leading to kidney toxicity	Inhibition, mitochondrial electron transport chain complexes	Occurrence, kidney toxicity	Baki Sadi		https://aopwiki.org/aops/437
447	Kidney failure induced by inhibition of mitochondrial electron transfer chain through apoptosis, inflammation, and oxidative stress pathways	Inhibition, mitochondrial electron transport chain complexes	Increased, kidney failure	Yann Gueguen		https://aopwiki.org/aops/447

*ACE* angiotensin-converting enzyme, *Ang-II* angiotensin II, *AO* adverse outcome, *AT1R* angiotensin-1 receptor, *MIE* molecular initiating event, *OECD* Organisation for Economic Cooperation and Development

**Fig. 1:**
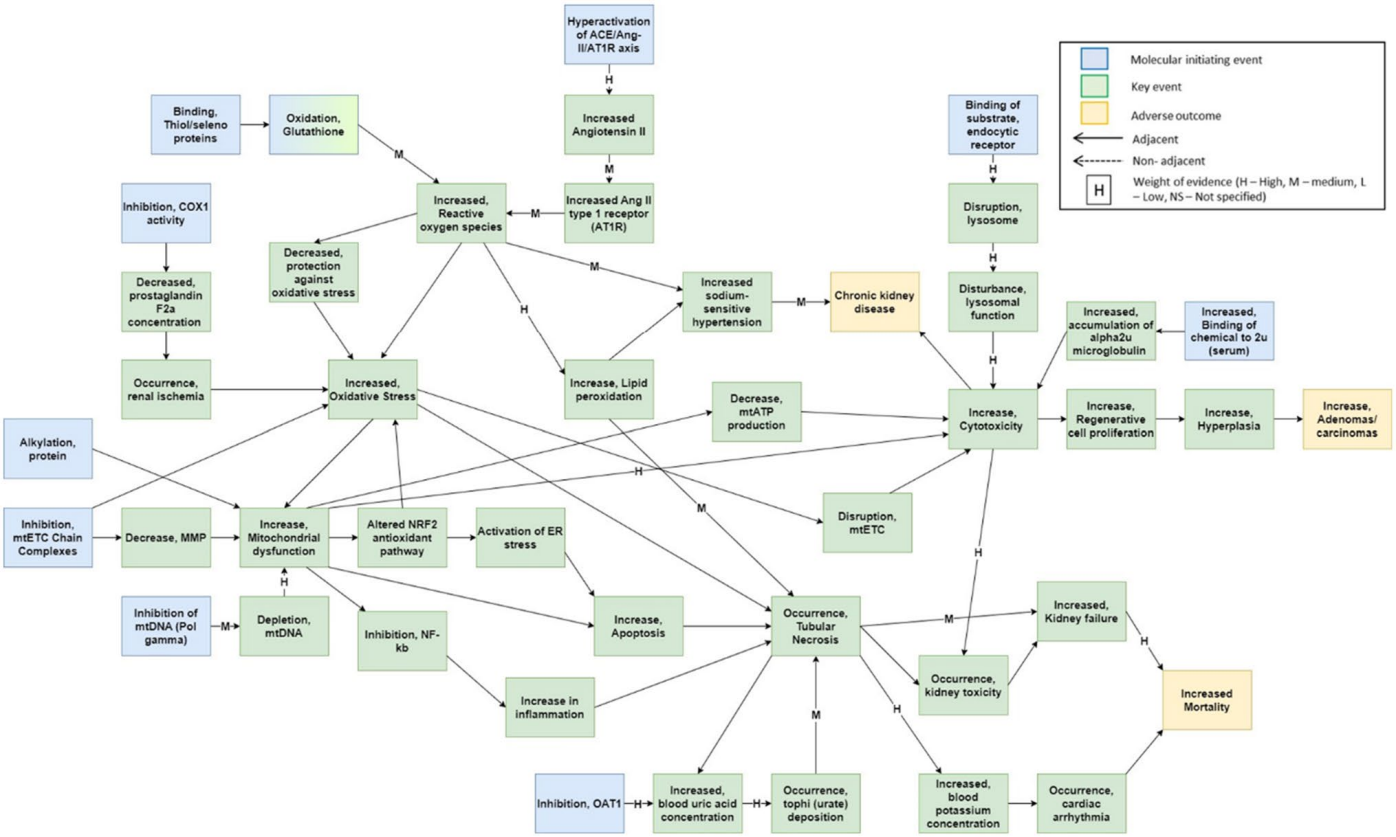
Network of 13 AOPs of nephrotoxicity available of on the AOP-Wiki containing adjacent key event relationships (extracted 1 May 2023). MIEs are denoted in blue, KEs in green and AOs in yellow. Solid arrows indicate adjacent KERs with arrow direction representing upstream to downstream KEs. KERs shared by multiple AOPs are represented by single arrows. The qualitative WoE between two KEs is annotated as either H (high), M (medium) or L (low). No label means there was a lack of information concerning the KER on the AOP Wiki. Curated KE titles, including abbreviations, have been included in the supplementary information

Relationships between KEs are typically represented as a linear pathway in AOP networks. However, it is also important to consider the potential for nonlinear or branching pathways, where a single KE may have multiple downstream influences, or else may interact with other KEs in different ways. For an accurate quantitative simulation of the AOP network, it is necessary for the network to depend on directly connected KEs. The distinction between adjacent and nonadjacent KERs was considered, resulting in the identification of a total of four nonadjacent KERs within two separate AOPs, including AOP 138 (OAT1 inhibition leading to renal failure and mortality) and AOP 186 (unknown MIE leading to renal failure and mortality). Following curation, however, it was noted that all nonadjacent KERs documented were described as adjacent in other AOPs and will be reported as such. From the selection of 13 individual AOPs outlined within Table 1, a nephrotoxicity AOP network (Fig. 1) was derived. This included both adjacent and nonadjacent KERs, as presented in the supplementary material (SI1), and drew from individual AOPs associated with AOs ranging from kidney toxicity and failure to chronic kidney disease (CKD) and the increased occurrence of adenomas/carcinomas. Furthermore, due its lack of contributory information towards a potential sequence of events leading to nephrotoxicity, the “Unknown, MIE” node was removed following construction of the network and not considered for all subsequent data analysis.

### Characterisation and analysis

**Fig. 2:**
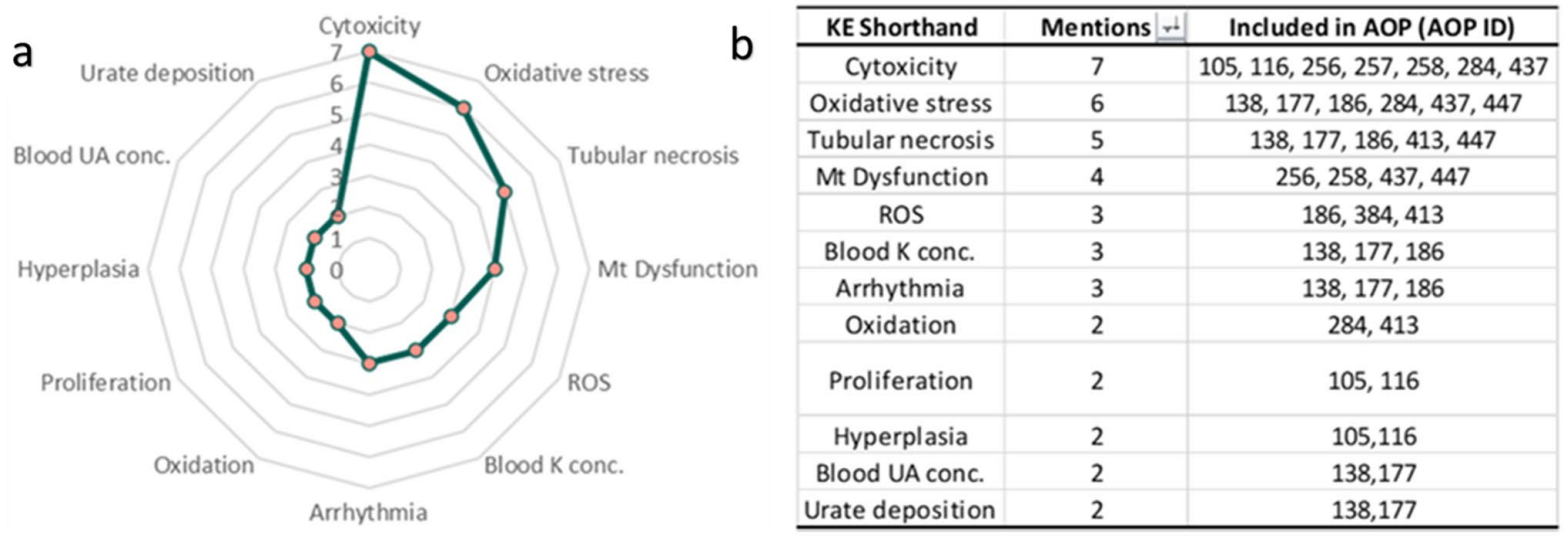
**a** Summary of KEs shared between multiple AOPs with a score of 2 or higher. The score displays the number of AOPs of which the KE is reported. Cytotoxicity had the highest score, meaning that it appeared in the most AOPs used to develop the network. **b** The distribution of KEs within the individual AOPs selected for the network

An examination of the nephrotoxicity AOP network yielded a total of 45 distinct KEs and their adjacent relationships (SI1). No KE was reported to appear in each of the 13 AOPs. The most frequently occurring KE (shared by 7 of the 13 AOPs included) was cytotoxicity. This was followed by oxidative stress, tubular necrosis, and mitochondrial dysfunction, which were each shared by six, five, and four AOPs, respectively (Fig. 2). Cytotoxicity was demonstrated to link the linear pathways of several common AOs, including CKD, kidney toxicity, kidney failure, the increased occurrence of adenomas and carcinomas, and increased mortality. Cytotoxicity was reported to serve as the MIE in AOP 116 (cytotoxicity leading to renal tubular adenomas and carcinomas in male rat) and was shown in other AOPs to be activated by factors including increased accumulation of alpha2u-microglobulin, disturbance of lysosomal function, decreased mitochondrial ATP (mtATP) production, disruption of the mitochondrial electron transport chain (mtETC), and increased mitochondrial dysfunction. KEs appearing downstream include kidney toxicity, increased regenerative cell proliferation, and CKD. In addition, the KEs oxidative stress and tubular necrosis were shared by four AOPs, of which either kidney failure or increased mortality was the AO.

**Fig. 3:**
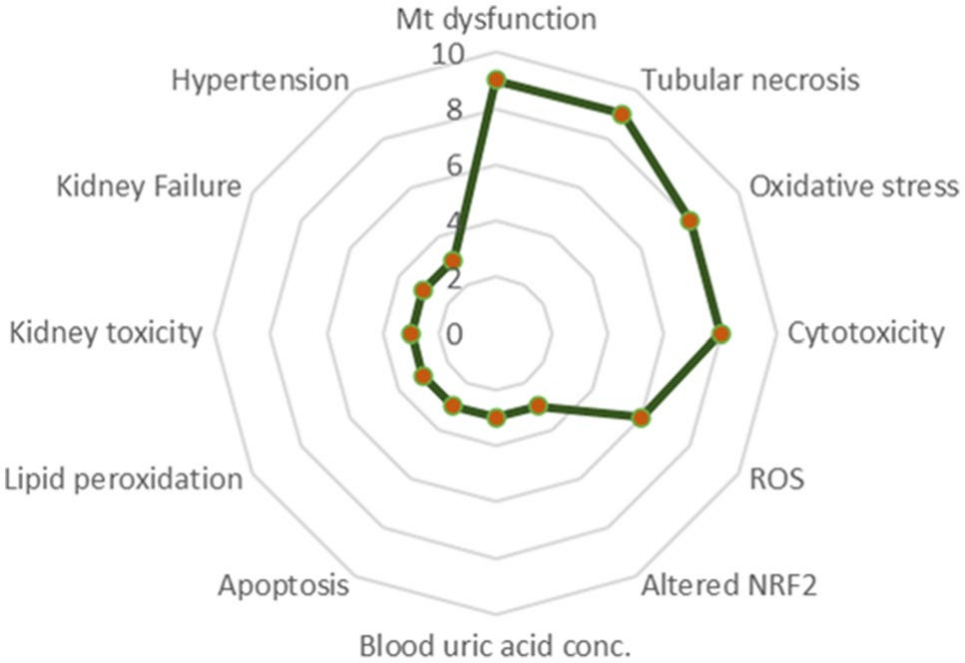
Summary of connectivity of KEs shared between multiple AOPs with a score of 3 or higher. The score displays the number of KERs associated the reported KEs from each AOP. Tubular necrosis had the highest score, meaning that it was the most interconnected KE among the network

Network analysis showed that, of all KEs, mitochondrial dysfunction and tubular necrosis possessed the highest degree of general connectivity—followed by cytotoxicity, oxidative stress, and increased ROS (with degrees of 9, 9, 8, 8, and 7, respectively). Conversely, the least connected KEs, with a degree of 1, were typically the MIEs from each selected AOP. A single AO, increased adenomas/carcinomas, also scored a degree of 1. A summary of the connectivity of those twelve KEs shared between multiple AOPs is depicted in Fig. 3. The level of KE connectivity within the network helps to determine key points of convergence and divergence (Table 2). Convergent KEs correspond to stages in the AOP network at which different pathways, ultimately sharing common toxic outcomes, are noted to meet. Divergent KEs, by contrast, represent stages whereby the downstream course of a particular pathway is liable to be altered by different factors (i.e. a fork point). Locating and understanding convergent and divergent KEs can aid in the identification and development of measurable in vitro and in silico strategies to prevent or mitigate adverse effects, as well as enhance understanding of the mechanisms underlying toxicity and disease.

**Table 2:** List of the identified 11 convergent and 13 divergent KEs

Convergent KEs		Divergent KEs	
KE type	KE name	KE type	KE name
KE	Occurrence, tubular necrosis	KE	Increase, Mt dysfunction
MIE/KE	Increase, cytotoxicity	KE	Increased, ROS
KE	Increase, oxidative stress	KE	Increase, lipid peroxidation
KE	Increased sodium-sensitive hypertension	KE	Altered NRF2 antioxidant pathway
KE/AO	Increased, kidney failure	MIE	Inhibition, mtETC complexes
KE/AO	Occurrence, kidney toxicity	MIE	Alkylation, protein
KE	Increase, apoptosis	MIE	Increased, Binding of chemicals to 2u
KE	Increased blood uric acid concentration	MIE	Inhibition, OAT1
AO	Chronic kidney disease	MIE	Binding of substrate endocytic receptor
AO	Increased mortality	MIE	Inhibition of mtDNA (Pol gamma)
AO	Increase, adenomas/carcinomas	MIE	Binding, thiol proteins/selenoproteins
		MIE	Inhibition, COX1 activity
		MIE	Hyperactivation of ACE/Ang-II, AT1R axis

*ACE* angiotensin-converting enzyme, *Ang-II* angiotensin II, *AO* adverse outcome, *AT1R* angiotensin-1 receptor, *COX1* cytochrome c oxidase 1, *MIE* molecular initiating event, *Mt* mitochondria, *mtDNA* mitochondrial DNA, *mtETC* mitochondrial electron transport chain, *NRF2* nuclear factor erythroid 2-related factor 2, *OAT1* organic anion transporter 1, *ROS* reactive oxygen species

Since the KERs in the AOP network are directional, the connectivity (degree) of an individual KE can be further specified—either as in-degree (i.e. denoting the quantity of incoming/downstream events) or as out-degree (quantity of outgoing/upstream events). The ratio of in-degree to out-degree values can be used to determine whether a KE is convergent or divergent. Degree scoring of the AOP network identified 11 convergent and 13 divergent KEs, where convergent KEs connect to more upstream than downstream KEs and divergent KEs connect to more downstream than upstream (Villeneuve et al. 2018). Tubular necrosis, oxidative stress, and cytotoxicity were identified as points of high convergence, each possessing in-degree scores of five. Thus, they were indicated to represent common and significant general features in nephrotoxicity. Conversely, mitochondrial dysfunction was a point of high divergence, having an out-degree score of five, highlighting its importance in the early onset of kidney disease aetiologies.

**Fig. 4:**
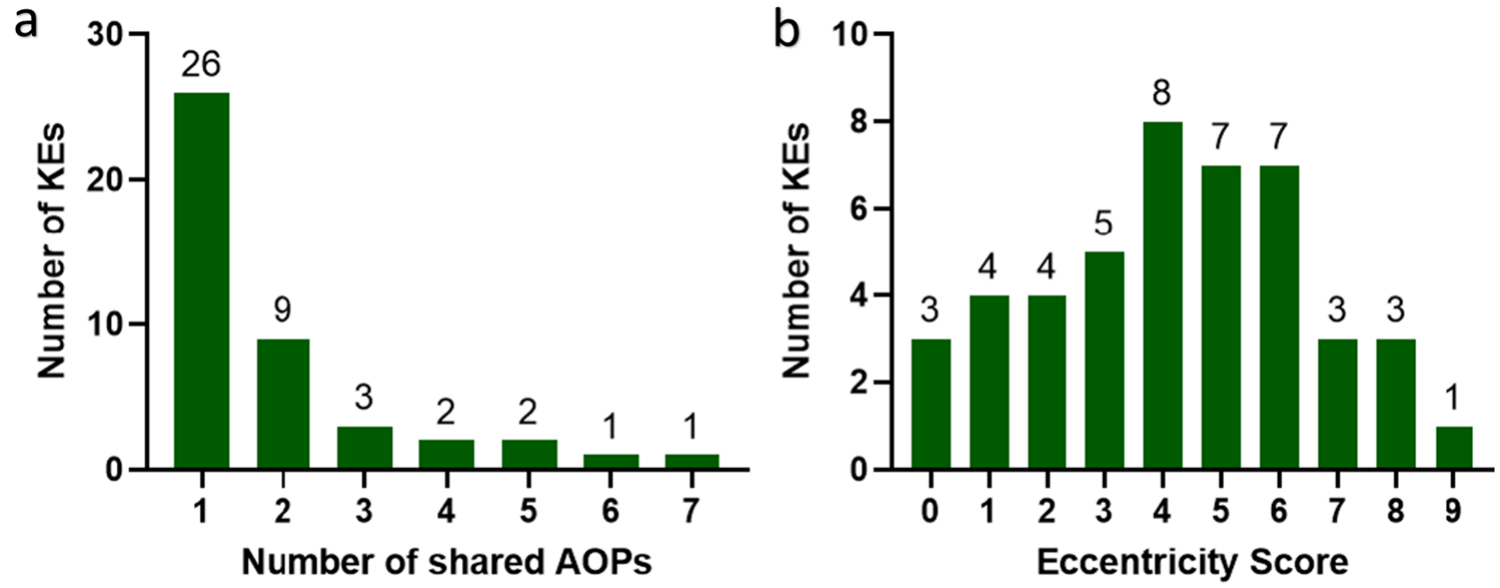
**a** KE distribution among shared AOPs show restricted interconnectivity within network. **b** KE distribution according to directed eccentricity score shows that 60% of KEs are unable to be characterised as either upstream or downstream due to their interconnectivity (eccentricity score between 3 and 6)

**Fig. 5:**
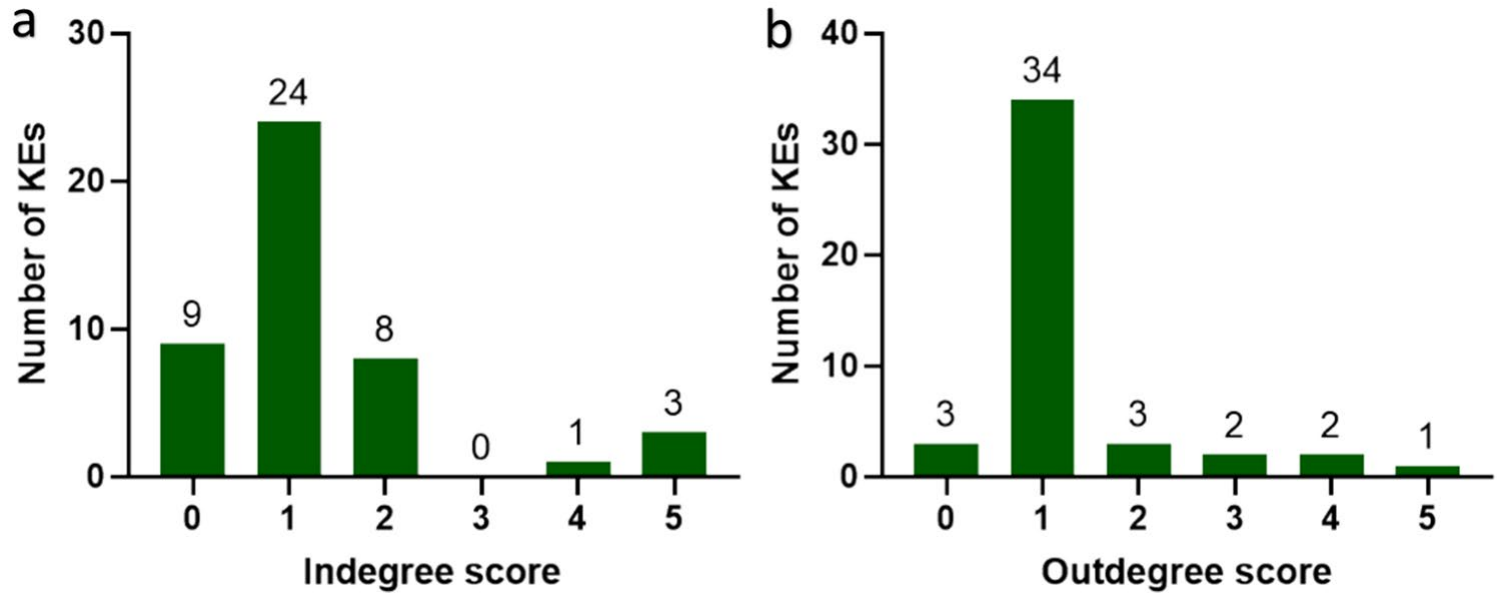
**a** The number of KEs and associated number of incoming KEs. **b** The number of KEs and associated number of outgoing KEs

Interestingly, despite being identified as a point of convergence, tubular necrosis had a high number of outgoing KERs, likely due to its association with extensive cell death ahead of acute kidney injury (AKI) and CKD. Similarly, mitochondrial dysfunction possessed a high number of incoming KERs despite being labelled as a divergent KE, thus indicating its crucial role within a variety of nephrotoxic adversities. All MIEs within the network were identified as points of divergence, except glutathione oxidation, which was connected to both an upstream and downstream KE. Glutathione is the main thiol-containing peptide involved in protecting against oxidative stress, and its oxidation can result in an increase in ROS downstream. However, it can also be activated upstream by the binding and activity of other thiol proteins/selenoproteins, such as thioredoxin reductase (https://aopwiki.org/aops/284). Eccentricity describes the centrality of a node (KE) within the network, by measuring the distance from that node to all other nodes within. Those with high eccentricity are further from most others, while those with low eccentricity are closer. The degree to which a KE is shared among AOPs varies from one to seven, but the interconnectivity of AOPs in the network is limited, since 58% of KEs are present in only one AOP (Fig. 4a). Additionally, based on directional eccentricity parameters, 24% of KEs were classified as occupying net upstream positions (eccentricity < 3) and 16% net downstream positions (eccentricity > 6). However, 60% of KEs could not be classified as either upstream or downstream owing to their greater interconnectivity (Fig. 4b). The statistical distribution of the number of KEs in relation to their in-degree and out-degree levels revealed that the majority within the network are connected only to one other—with 53% of in-degree KEs (Fig. 5a) and 76% of out-degree KEs demonstrating this pattern (Fig. 5b).

The development and analysis of a network can be influenced by the number of AOPs sharing a given KE, since network modelling is improved when KEs are shared between at least two individual AOPs. Sharing a KE across multiple AOPs enhances network reliability by validating its importance for multiple AOs, reducing reliance on a single pathway. Betweenness centrality is a measure of node centrality within a network, quantifying the extent to which a KE may be seen to bridge the shortest path between two other KEs. This metric can be used to identify events that are particularly influential within a network. However, in this instance, the related but modified parameter of AOP simple path occurrence (as described within Sect. “Network analysis”), was adopted. Through it, the events of increased cytotoxicity, tubular necrosis, oxidative stress, ROS and mitochondrial dysfunction (with respective scores of 0.514, 0.443, 0.414, 0.386 and 0.329) were highlighted as most central.

This information supports the results given by the degree scoring and conforms to the assortment of KEs in the graphical representation of the AOP network. An overview of available assays for measuring endpoints and a selection of stressors associated with these five KEs are presented in Table 3.

**Table 3:** Overview of assays that can be used to measure endpoints associated with the three most highly connected and central KEs in the nephrotoxicity AOP network

Key event	Stressors	Endpoint	Assay/method	Methodology	References
Tubular necrosis /cytotoxicity	Cisplatin, gentamicin, doxorubicin, cadmium	Cell death/necrosis	Prestoblue	Fluorescent measurement of resazurin reduction by metabolically active cells	Lall et al. (2013)
			LDH release assay	Colorimetric assay measuring LDH released into culture medium	Kabakov and Gabai (2018)
		Apoptosis	Caspase 3/7 activity assay	Fluorometric assay detecting fluorogenic substrate cleaved by caspase 3/7	
			TUNEL assay	Detection of fragmented DNA using fluorescent probes	
Mitochondrial dysfunction	Cisplatin, tenofovir, gentamicin, vancomycin	Mitochondrial membrane potential (MMP)	JC-1/JC-10 assay	Changes in ratio of red/green fluorescence of polarised mitochondria using fluorescent probes	Sivandzade et al. (2019)
		Mitochondrial respiration	Oxygen consumption rate (OCR) assay	Measurement of the rate of oxygen consumption using fluorescent probes	Plitzko and Loesgen (2018)
		Mitochondrial ROS (mtROS)	MitoSOX, MitoPY1, or MitoTracker Red	Detection of mtROS using fluorescent probes	Chen and Mathews (2014)
Oxidative stress	Cisplatin, gentamicin, cadmium	ROS levels	ROS	Measuring fluorescence of dihydroethidium or fluorescein derivatives	
		Redox status	GSH/GSSG ratio	Measurement of the ratio of reduced glutathione (GSH) to oxidised glutathione disulphide (GSSG)	Owen and Butterfield (2010)
		Lipid peroxidation	MDA assay	Spectrophotometric measurement of reacting MDA with a chromogenic reagent	Aguilar Diaz De Leon and Borges (2020)
			TBARS assay	Identifying the presence of MDA by reacting it with TBARS	

*GSH* glutathione, *GSSG* glutathione disulphide, *LDH* lactate dehydrogenase, *MDA* malondialdehyde, *MMP* mitochondrial membrane potential, *mtROS* mitochondrial ROS, *OCR* oxygen consumption rate, *ROS* reactive oxygen species, *TBARS* thiobarbituric acid-reactive substances, *TUNEL* terminal deoxynucleotidyl transferase (TdT) dUTP nick-end labelling

**Fig. 6:**
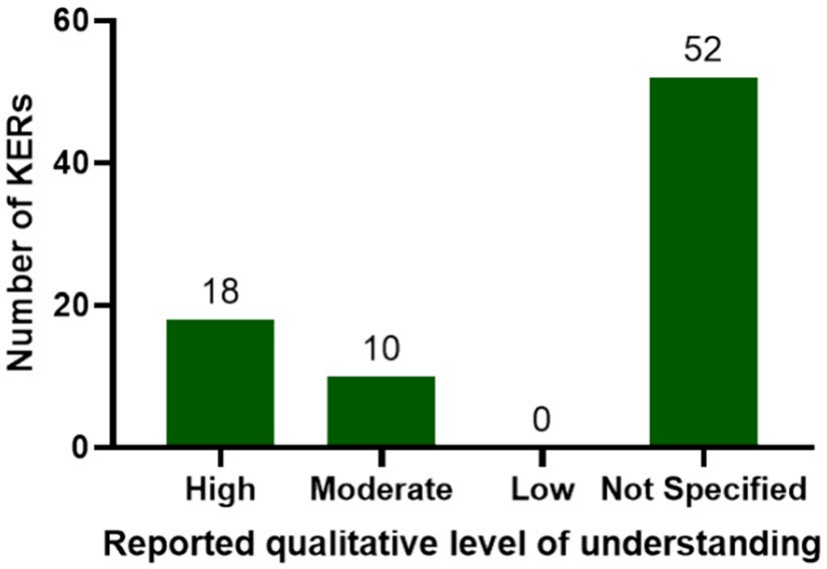
Distribution of the weight of evidence for AOP KERs as reported by the developers

Previously, a comprehensive network was designed in order to analyse the connectivity of existing AOPs present on the AOP-Wiki (Pollesch et al. 2019). However, it was found that the majority of AOPs submitted lacked information in user-defined fields. Out of all KERs in the network, 35% were reported to have either a moderate (13%) or a higher (23%) level of qualitative understanding, as determined by individual AOP developers (Fig. 6). Interestingly, a majority of the KERs with high understanding (10 from 18) are present within just three of the adopted AOPs. For many AOPs (covering all 65% of reported KERs), the qualitative level of KER understanding remains unspecified. It is worth noting that AOPs submitted to AOP-Wiki may be at very different stages of development. Some are not yet finalised, thus ensuring that the level of understanding for reported KERs often remains unreported. In this case, all AOPs that recorded unspecified levels of qualitative KER understanding were reported to still be under development. All AOPs, regardless of their developmental stage, were nevertheless included in the characterisation and analysis of the network.

## Discussion and conclusions

The present study utilised an established method (Arnesdotter et al. 2021; Spinu et al. 2019) to construct and examine an AOP network for nephrotoxicity. Although the network may not encompass all mechanisms of nephrotoxicity, this exercise nevertheless provided valuable direction for prioritising KEs for purposes of testing. Except in highly specific cases, AOP networks can serve as predictive functional units for most chemically induced AOs (Knapen et al. 2018). Thus, it is reasonable to focus on KEs shared across multiple AOPs relevant to nephrotoxicity. Analysis of the network identified tubular necrosis, mitochondrial dysfunction, and oxidative stress as the most connected and central KEs. Therefore, these should be considered during the selection, development, and optimisation of in vitro or in silico assays for predicting chemical-induced nephrotoxicity. However, it is important to note that consideration should also be given to the application of all appropriate in vitro assays, in order to combat potential redundancies when establishing future models. Cytotoxicity was the most reported KE in the network, based on the prerequisites defined previously. However, due to the assumed commonality of this KE across AOPs describing various biological systems, it was not judged sufficiently unique to warrant exclusive consideration for in vitro assay development relating to nephrotoxicity. Instead, it is recommended that testing of this KE be combined or substantiated with additional, more disease-specific endpoints.

Network analysis showed that kidney toxicity was commonly caused by increased cytotoxicity and tubular necrosis, leading ultimately to increased mortality. Chemical-induced tubular necrosis can occur via several upstream mechanisms, including oxidative stress, inflammation, lipid peroxidation, ischaemia through vasoconstriction, and urate deposition. Perturbations of this KE can trigger downstream processes, including increased blood potassium and uric acid concentrations, kidney toxicity, and kidney failure. Oxidative stress and mitochondrial dysfunction were also identified as primary upstream KEs. Oxidative stress can emerge from several KEs, including alterations to the nuclear factor erythroid 2-related factor 2 (Nrf2) antioxidant pathway, the increased general production of reactive oxygen species (ROS), and kidney ischaemia (Chazelas et al. 2021; Ma 2013). In turn, this may lead to downstream mitochondrial dysfunction (incorporating ETC disruption) and tubular necrosis. Mitochondrial dysfunction can further manifest through decreased mitochondrial membrane potential (MMP), depletion of mitochondrial DNA (mtDNA), and the alkylation of key proteins (Lax et al. 2011; Zorova et al. 2018). Downstream influences of mitochondrial dysfunction included inhibition of NF-κβ, increased apoptosis, decreased mtATP production, and increased cytotoxicity. It was also shown to be part of a feedback loop with oxidative stress and alterations to Nrf2 signalling. Increased ROS, blood potassium concentrations, and cardiac arrhythmia were shared by three AOPs, whereas glutathione oxidation, increased hyperplasia, blood uric acid concentration, urate deposition, and increased regenerative cell proliferation were shared by two. Inhibition of the mtETC complexes was the only MIE to be shared by multiple AOPs, all leading to kidney toxicity.

Nonadjacent KERs can help in identifying branching pathways and areas of potential crosstalk between different AOPs by detailing the causal relationship between KEs that occur relatively distant from one another (Villeneuve et al. 2018). Nonadjacent KERs have been reported to be associated with multiple biological processes and have helped to identify steps in the causal chain that can be targeted for intervention to prevent or mitigate the AO (Spinu et al. 2019). The incorporation of both adjacent and nonadjacent KERs in AOP networks implies the presence of additional connections and increasing biological complexity. Furthermore, it may aid in defining the supporting WoE, while simultaneously preserving the modular network layout. However, this may also influence network parameters—leading to increased node degree and AOP simple path occurrence values or to decreased eccentricity (Villeneuve et al. 2018). It should be noted that two events labelled as nonadjacent under an individual AOP may become “bridged” upon integration into a network. This is possible in instances where the very same sequence is found described through an alternative pathway, albeit with the presence of intermediate, adjacent, linking steps. The AOP network developed in the present study exhibited limited interconnectivity, likely due to the diverse mechanisms involved in nephrotoxicity (Kim & Moon 2012; Kwiatkowska et al. 2021). As such, some pathways did not pass through the common KEs, possibly due to differences in reported detail among the constituent AOPs. It is important to note that the “AOP mining” method used was restricted to available information on AOP-Wiki, leading to potential coverage gaps and incomplete information as regards certain pathways. Therefore, not all AOPs from the literature may have been included. The lack of validated AOPs related to nephrotoxicity also posed a significant challenge. However, these points should not be seen as a direct criticism of their developers, but rather as highlighting areas that are in need of improvement for the effective utilisation of AOPs. While the AOP-Wiki serves as an invaluable resource for the advancement and dissemination of knowledge concerning AOPs, its inherent reliance on user-uploaded information impacts the precision and dependability of the content displayed on the platform. For example, AOP 105 (alpha_2u_-microglobulin cytotoxicity leading to renal tubular adenomas in male rat) and AOP 116 (cytotoxicity leading to renal tubular adenomas and carcinomas in male rat) were connected to the network by the cytotoxicity KE, albeit without inclusion of details relating to the exact contributory mechanisms underlying its emergence. While proximal tubule toxicity was identified as the common KE, indication as to any additional events occurring at the molecular or cellular level was not given.

The AOP framework considers both empirical evidence and the biological plausibility of KERs to capture the WoE for causal linkages (Becker et al. 2017; Villeneuve et al. 2014). In this regard, AOPs are generally constructed based on empirical data that describe the biological pathway in one or in several species. While the developers may presume that the biological plausibility of an AOP implies a broad taxonomic domain of applicability, supporting evidence is often drawn from only a limited species range. Defining the extent of taxonomic applicability is of great importance, since it helps to ensure confidence in the relevance and utility of the AOP across varying organisms and settings (OECD 2018b). However, different species often have diverse physiological and biochemical characteristics that serve to affect how they respond to chemical exposure, thus presenting a challenge when attempting to extrapolate these outcomes to human populations. For example, AOP 138 (OAT1 inhibition leading to renal failure and mortality) and AOP 177 (COX1 inhibition leading to renal failure and mortality) were developed utilising data drawn from various bird species. While the kidneys of humans and birds are similar in function, there are significant differences in their anatomy and physiology, meaning that the results of nephrotoxicity studies conducted in one may not accurately reflect the effects of a particular substance on the other.

Similarly, AOP 413 (oxidation of reduced glutathione leading to mortality) focussed on investigating the mechanisms of uranium toxicity for acute renal failure in fish species, with a scope limited exclusively to consideration of events occurring following exposure in aqueous media. Yet, despite the recent emergence of zebrafish as a model to study kidney function and disease (Outtandy et al. 2019), the kidneys of fish and humans also differ in structure and function; fish kidneys are less complex and lack the same level of specialisation. However, in this particular case, an article expanding on AOP 447, with a focus on the applicability of uranium toxicity to human health has been published—referencing several reports reviewing informative cases of acute human exposure to uranium (Gueguen & Frerejacques 2022). They expand on how observed alterations in kidney biomarkers for individuals who have been overexposed to uranium may be attributed to tubular necrosis, which was deemed the most significant clinical outcome of acute exposure in humans. By considering taxonomic applicability during development, researchers may ensure that an AOP can be used with confidence to predict potential adverse effects across different species. If an AOP is only applicable across a narrow range of taxa, then it may be necessary to conduct additional studies to fill data gaps within other species. However, the approach utilised for the development of this AOP network helped to provide a means to compare the results of two distinct AOPs, thus simultaneously enabling the identification of similarities in nephrotoxic responses, while preserving the species-specific findings for each.

AOPs should be chemical-agnostic since they aim to provide a general framework for understanding the biological pathways and potential AOs associated with a MIE and its downstream effects, regardless of the specific chemical involved (Vinken et al. 2017). This allows for greater flexibility in applying the AOP framework across a range of chemicals and chemical classes, as well as the ability to identify commonalities in pathways that lead to AOs. Although AOPs are not limited to specific chemicals, they can still be employed to facilitate the application of a mode (and/or mechanism) of action framework in understanding the harmful impacts of established stressors. In brief, the term “stressor” is used to refer to agents (either chemical or non-chemical) which are associated with the initiation of a given AO. Proposing stressors is important in AOP development, as it can help towards identifying specific mechanisms and pathways involved in the incidence of the AO (Aguayo-Orozco et al. 2019). Yet, only six of the thirteen AOPs used to develop this network have listed stressors in their respective submissions to the AOP-Wiki—despite it being reasonable to assume that most supporting empirical evidence would have been derived from the chemical stressors affecting them. Likewise, quantitative KERs established using these stressors can introduce elements of uncertainty, given that chemicals often exert their effects through multiple mechanisms or pathways (Perkins et al. 2019). Proper identification of appropriate stressors will be important for establishing a foundation of knowledge that can be built upon in the future development of robust AOPs. Without it, the AOP may be considered incomplete or inaccurate and lacking in specificity, thus making it difficult to establish causal relationships between exposure and the AO.

AOPs and their networks provide a framework to organise and integrate information on the mechanisms by which chemicals or stressors lead to kidney toxicity and failure. This can help identify KEs and biological pathways that are important in the development of nephrotoxicity. AOPs can also help in guiding the selection of appropriate in vitro assays to detect and assess nephrotoxicity and thus provide a mechanistic basis for interpreting their results. Moreover, AOPs can aid in the development of novel testing strategies and risk assessment approaches, such as in silico models and read-across methods (Pletz et al. 2018; Vinken et al. 2021). Applications of the individual AOPs used to derive the network have varied, between continued development and WoE assessment, through to the evaluation of published literature (Goyak et al. 2022; Gueguen and Frerejacques 2022), and the formulation of a proof of concept for the viability of an in vitro-based risk assessment via integration of mechanistic endpoints and toxicokinetic modelling (Jarzina et al. 2022). The AOP network established in this study provides a comprehensive and advanced mechanistic representation of nephrotoxicity, detailing connections between multiple pathways and adverse effects, including kidney failure and CKD. The OECD AOP-Wiki serves as an exceptional database for curating, assessing, and authenticating linear AOPs. It not only ensures the reliability of mechanistic data, but also aids in identifying gaps in knowledge and prioritising testing techniques. By providing a more comprehensive understanding of the mechanisms underlying nephrotoxicity, AOP networks can contribute to more accurate predictions of the potential hazards and risks of chemicals and ultimately improve the protection of human health and the environment.

### Supplementary Information

Below is the link to the electronic supplementary material.

Supplementary file1 (XLSX 44 KB)

## Data Availability

The authors confirm that the data supporting the findings of this study are available within the article and its supplementary materials.

## Abbreviations

5-HT_2C_: 5-Hydroxytryptamine receptor 2C
AKI: Acute kidney injury
AO: Adverse outcome
AOP: Adverse outcome pathway
COX: Cyclooxygenase
GSH: Glutathione
GSSG: Glutathione disulphide
KE: Key event
KER: Key event relationship
LDH: Lactate dehydrogenase
MDA: Malondialdehyde
MIE: Molecular initiating event
MMP: Mitochondrial membrane potential
mtATP: Mitochondrial adenosine triphosphate
mtDNA: Mitochondrial DNA
mtETC: Mitochondrial electron transport chain
mtROS: Mitochondrial ROS
NF-κβ: Nuclear factor kappa-light-chain-enhancer of activated B cells
Nrf2: Nuclear factor erythroid 2-related factor 2
OAT: Organic anion transporter
OCR: Oxygen consumption rate
OECD: Organisation for Economic Co-operation and Development
QSAR: Quantitative structure–activity relationship
ROS: Reactive oxygen species
TBARS: Thiobarbituric acid-reactive substances
TUNEL: Terminal deoxynucleotidyl transferase (TdT) dUTP nick-end labelling
WoE: Weight of evidence
